# The Diagnostic Value of Pan-Trk Expression to Detect Neurotrophic Tyrosine Receptor Kinase (NTRK) Gene Fusion in CNS Tumours: A Study Using Next-Generation Sequencing Platform

**DOI:** 10.3389/pore.2022.1610233

**Published:** 2022-02-28

**Authors:** Fawaz Mohamed, Maher Kurdi, Saleh Baeesa, Abdulrahman Jafar Sabbagh, Sahar Hakamy, Yazid Maghrabi, Mohammed Alshedokhi, Ashraf Dallol, Taher F. Halawa, Ahmed A. Najjar, Imad Fdl-Elmula

**Affiliations:** ^1^ Department of Pathology, Faculty of Medicine, King Abdulaziz University, Rabigh, Saudi Arabia; ^2^ Neuromuscular and Brain Tumour Unit, King Abdulaziz University, Jeddah, Saudi Arabia; ^3^ Division of Neurosurgery, Department of Surgery, Faculty of Medicine, King Abdulaziz University, Jeddah, Saudi Arabia; ^4^ Department of Neuroscience, King Faisal Specialist Hospital, Jeddah, Saudi Arabia; ^5^ Centre of Excellence of Genomic Research, King Abdulaziz University, Jeddah, Saudi Arabia; ^6^ Department of Pediatrics, Faculty of Medicine, King Abdulaziz University, Rabigh, Saudi Arabia; ^7^ College of Medicine, Taibah University, Almadinah Almunawwarah, Saudi Arabia; ^8^ Department of Clinical Genetics, Faculty of Medicine, Al-Neelain University, Khartoum, Sudan

**Keywords:** immunohistochemistry, next-generation sequencing, CNS tumours, NTRK-fusions, Pan-Trk, TruSight Oncology500

## Abstract

**Background:** Neurotrophic tyrosine receptor kinase (*NTRK*) fusion has been detected in rare types of CNS tumours, which can promote tumorigenesis. The efficacy of Trk inhibitor became a significant therapeutic interest. Our aim was to investigate whether Pan-Trk immunohistochemistry (IHC) is a reliable and efficient marker for detecting *NTRK-*fusion in different brain tumours.

**Methods:** This study included 23 patients diagnosed with different types of CNS tumours. Testing for Pan-Trk IHC with monoclonal Ab (EPR17341) has been performed on all FFPE tissues. Parallelly, *NTRK*-rearrangements were tested using both DNA and RNA-based next-generation sequencing (NGS) assay using TruSight Onco500 platform.

**Results:** The cohort included eight pilocytic astrocytomas, one oligodendroglioma, six IDH^wildtype^ glioblastomas, four IDH^mutant^ grade four astrocytomas, and one sample of each (astroblastoma, central neurocytoma, medulloblastoma, and liponeurocytoma). The mean age was 35 years; seven cases were in the paediatric age group, and 16 were adult. Pan-Trk expression was detected in 11 (47.8%) tumours, and 12 (52.1%) tumours showed no Pan-Trk expression. Nine Cases (82%) with different Pan-Trk expressions did not reveal *NTRK*-rearrangement. The other two positively expressed cases (liponeurocytoma and glioblastoma) were found to have *NTRK2*-fusions (*SLC O 5A1-NTRK2, AGBL4-NTRK2, BEND5-NTRK2*). All the 12 cases (100%) with no Pan-Trk expression have shown no *NTRK*-fusions. There was no statistically significant association between Pan-Trk expression and *NTRK*-fusion (*p* = 0.217). The detection of *NTRK-* fusions using NGS had high specificity over *NTRK*-fusion detection by using Pan-Trk IHC.

**Conclusion:** Pan-Trk IHC is not a suitable tissue-efficient biomarker to screen for *NTRK*-fusions in CNS tumours, however RNA-based NGS sequencing should be used as an alternative method.

## Introduction

**FIGURE 1:**
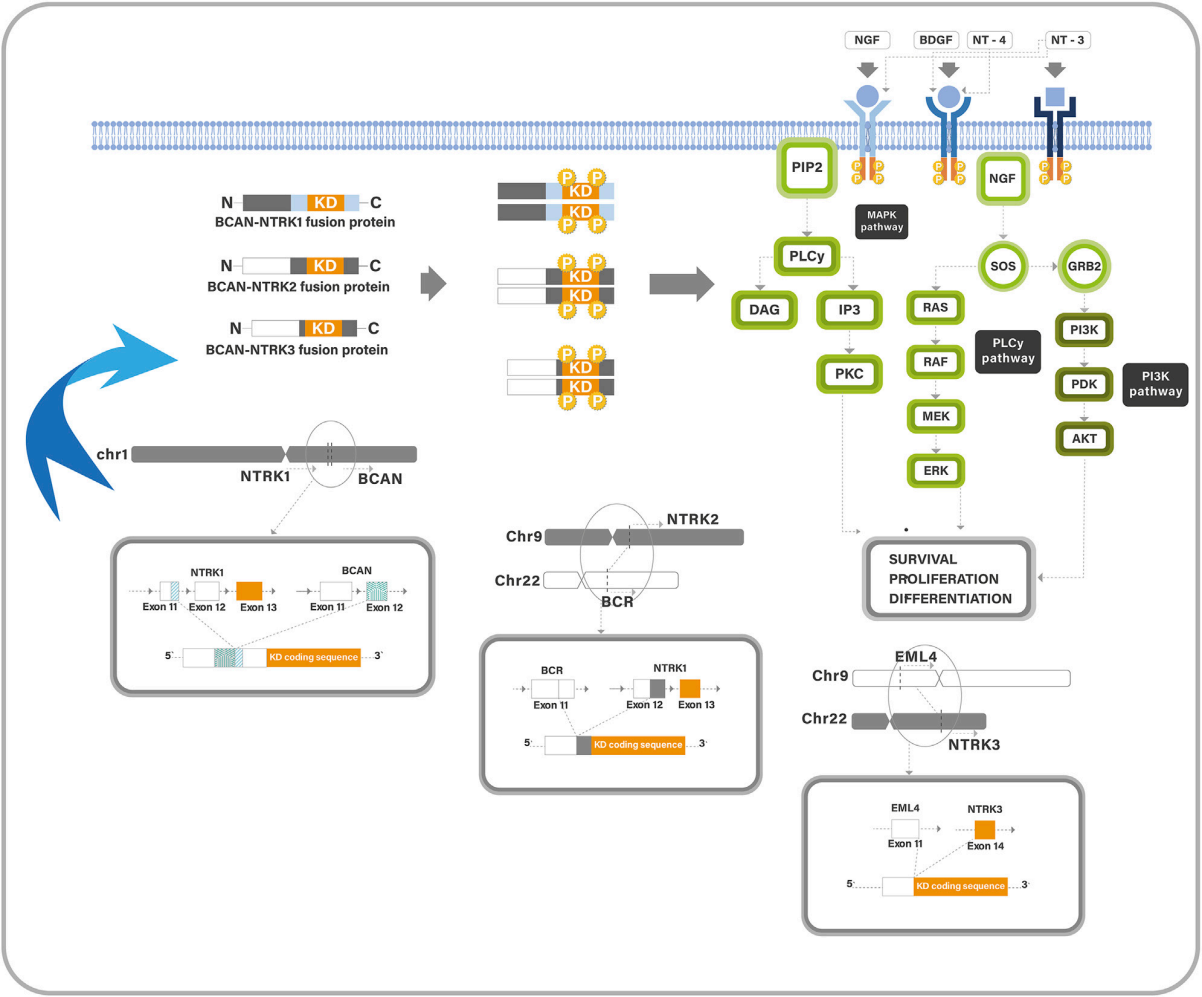
A diagram describes the Trk pathway and the oncogenic mechanism of *NTRK*-fusions. Trk proteins contain an intracellular TK domain which promotes cell proliferation through MAPK/ERK, PLCg/PKC, and PI3K/AKT pathways. Trk-fusion proteins have a complete TK domain, and the partner gene is expressed in a homodimer, which induces ligand-independent activation of the TK domain. It also activates the cancer-associated pathways.

The neurotrophic tyrosine receptor kinase (*NTRK*) is a family member of three genes (*NTRK1, NTRK2, NTRK3*), which produce tyrosine kinase (TK) proteins (Trk-A, Trk-B, and Trk-C) (1). Their receptors are highly expressed in neural tissue, in which they play an important role in neuronal development, proliferation, synaptic plasticity, and cognition and memory (2). They have a similar structure; each consists of an extracellular ligand-binding domain, a transmembrane region, and an intracellular kinase domain. Each receptor has a ligand that it prefers; Trk-A has the highest affinity for neurotrophin nerve growth factor, Trk-B for brain-derived neurotrophic factor and neurotrophin-4, and Trk-C for neurotrophin-3 (1). Normally, ligands binding to the extracellular region may stimulate the kinase domain of the Trk receptor, resulting in homodimerization, phosphorylation, and activation of signaling pathways (Figure 1). Rearrangements in the *NTRK* gene can result in two genes fusing at the C-terminal TK-domain with N-terminal fusion partner producing altered Trk proteins. This fusion may lead to uncontrolled growth of tumour cells (1). Fusions involving the *NTRK* genes can be oncogenic drivers, leading to abnormal Trk receptor dimerization and constitutive activation of Trk pathways, resulting in upregulation and apoptosis resistance (2).

The prevalence of *NTRK*-fusion is less than 1% of all tumors. It can be found in approximately 1,500–5,000 children and adults with cancers annually (3). It has been identified in a broad range of solid tumours in adults, including salivary gland cancer, thyroid cancer, breast cancer, gastrointestinal cancers, gynecological cancers, non-small cell carcinoma of lung, and some soft tissue sarcomas (3–5). In children, these fusions have been identified in rare cases of diffuse CNS gliomas as well as in melanoma, soft-tissue sarcomas, inflammatory myofibroblastic tumours, congenital infantile fibrosarcoma, and mesoblastic nephroma (4). About 0.55–2% of all neuroepithelial tumours and gliomas contain *NTRK*-fusions (2).

Hechtman et al. detected four of 23 glioblastomas with *NTRK*-fusion (6). Torre et al. tested *NTRK*-fusions in 42 patients with CNS glioma of different ages. *NTRK*-fusion was detected in CNS gliomas of the infants (7 cases), adult cases (22 cases), and pediatric cases >1 year (13 cases). Most pediatric cases had *NTRK2-*fusions (69%, 9 cases), while *NTRK1-*fusions were found in most adult glioma cases (68%, 15 cases) (2).

Due to the efficacy of food and drug administration (FDA) approved *NTRK* targeted therapy in non-CNS cancers, it is important to identify patients with *NTRK*-fusion driven brain tumours using the accurate technique (1, 2). *NTRK-*fusions are generally detected at the molecular level, using fluorescent *in-situ* hybridization technique (FISH) or preferably a next-generation sequencing (NGS) of targeted DNA or RNA testing. However, molecular studies are still costly and time-consuming, and technical risks such as nucleic acid degradation might arise. Alternatively, using the immunohistochemistry (IHC) technique, Pan-Trk staining is typically less expensive, has a rapid turnaround time, and is more tissue-efficient for *NTRK*-fusion detection (6). Pan-Trk IHC expression was commonly used in non-CNS cancers because of its high specificity and sensitivity. Solomon et al. found that Pan-Trk was strongly expressed in lung, pancreatic, colorectal, thyroid, and biliary carcinomas (1). The specificity decreased in salivary gland and breast carcinomas as well as soft tissue sarcomas with neural or smooth muscle differentiation (1). Hechtman et al. has detected some glioblastomas with *NTRK*-fusions, which were compatible with the IHC staining and Archer RNA test (6). It was concluded that the sensitivity of IHC with Pan-Trk in their cohort was 95%, and the specificity was 100% for *NTRK-*fusions (6). At the same time, Solomon et al. found that the sensitivity for *NTRK1* was 96% and for *NTRK2* was 100%, while sensitivity for *NTRK3* fusions was 79% (7). It was clear that Pan-Trk staining variability correlates with fusion partners.

Our study assessed Pan-Trk expression using IHC technique in different types of CNS tumours, and we tested its specificity, sensitivity, and accuracy with NGS technique. In addition, we explored if Pan-Trk expression can be used as a reliable biomarker immunolabeling to detect *NTRK*-fusions in CNS tumours.

## Materials and Methods

### Patients’ Stratification

This study included 23 patients, aged between 3 and 64 years and histologically diagnosed as eight cases of pilocytic astrocytomas, one case of oligodendroglioma, six cases IDH^wildtype^ glioblastomas, four cases IDH^mutant^ WHO grade four astrocytomas, and one case of each (astroblastoma, central neurocytoma, medulloblastoma, and liponeurocytoma) (Table 1). The study was approved by the National Biomedical Ethics Committee at King Abdulaziz University (HA-02-J-008) under a general ethical report. Patients’ clinical data were retrieved from hospital records and included patients ‘age at diagnosis, gender, tumour location and type, 2021 WHO grading, and IDH1 mutational status (Table 1). The histological diagnosis was made based on 2021 WHO classification of CNS tumours (8, 9).

**TABLE 1:** Demographic data of the 23 patients with CNS tumours, including NGS findings of DNA-based and RNA-based mutations.

Age	Gender	Location	Tumour	Grade	PanTrk	LI (%)	DNA-based mutation	RNA-based mutation	TMB	MSI	NTRK	IDH
28	Male	Frontal	Liponeurocytoma	II	Diffuse	90	None	SLCO5A1-NTRK2	low	stable	Detected	Not done
10	Male	Posterior fossa	Pilocytic astrocytoma	I	Diffuse	90	BRAFV600E/TP53	None	medium	none	Not detected	Not done
54	Male	Parietal	Astrocytoma	IV	No	0	IDH1/ATRX/TP53/BCOR/PTCH1	EGFR amplification	medium	stable	Not detected	mutant
33	Male	Frontal	Oligodendroglioma	III	No	0	MET	None	medium	stable	Not detected	mutant
40	Male	Temporal	Astroblastoma	None	No	0	BRAFV600 E	None	low	stable	Not detected	wildtype
8	Male	Posterior fossa	Pilocytic astrocytoma	I	No	0	None	AUTS2-BRAF/PRKAR2B-BRAF	low	stable	Not detected	Not done
52	Male	Frontal	Glioblastoma	IV	Partial	40	TERT/PTEN/FGFR4	AGBL4-NTRK2/BEND5-NTRK2	low	stable	Detected	wildtype
53	Male	Frontal	Glioblastoma	IV	No	0	TP53	None	high	stable	Not detected	wildtype
4	Female	Spinal	Pilocytic astrocytoma	I	No	0	None	None	medium	none	Not detected	Not done
62	Male	Parietal	Glioblastoma	IV	No	0	BRAFV600E/TP53/APC	None	low	stable	Not detected	wildtype
53	Male	Temporal	Pilocytic astrocytoma	I	Focal	10	TERT/	EGFR amplification/CD4-6 gain	low	stable	Not detected	wildtype
45	Female	Parietal	Glioblastoma	IV	Diffuse	85	PTEN	CDK6 gain/EGFR amplification	low	stable	Not detected	wildtype
40	Male	Posterior fossa	Pilocytic astrocytoma	I	Focal	8	BRAFV600 E	None	medium	stable	Not detected	Not done
6	Female	Cerebellar	Medulloblastoma	IV	Diffuse	90	PTCH1	CDK6 gain	low	stable	Not detected	Not done
3	Female	Frontal	Pilocytic astrocytoma	I	Focal	10	None	None	medium	stable	Not detected	Not done
36	Male	Lateral ventricle	Central neurocytoma	II	No	0	None	None	low	stable	Not detected	Not done
45	Male	Temporal	Glioblastoma	IV	No	0	TERT	None	medium	stable	Not detected	wildtype
12	Female	Posterior fossa	Pilocytic astrocytoma	I	No	0	FGFR1	None	low	stable	Not detected	Not done
14	Male	Hypothalamic	Pilocytic astrocytoma	I	No	0	BARD1	KIAA1549-BRAF	low	stable	Not detected	Not done
64	Female	Temporal	Glioblastoma	IV	Focal	10	ATRX/TERT	None	medium	none	Not detected	wildtype
51	Female	Frontal	Astrocytoma	IV	Partial	45	PTEN	None	high	stable	Not detected	mutant
60	Male	Temporal	Astrocytoma	IV	Partial	40	IDH1	None	medium	none	Not detected	mutant
51	Female	Parietal	Astrocytoma	IV	Non	0	IDH1	None	high	stable	Not detected	mutant

LI, labelling index; TMB, tumour-burden; MSI, microsatellite instability.

### Tumour Samples

Archival routine formalin-fixed and paraffin-embedded (FFPE) tumour tissues were collected from 23 patients with different CNS tumours. Haematoxylin and Eosin (H&E)-stained sections were re-examined by a certified neuropathologist (MK) to confirm that the histopathological diagnosis was made based on 2021 WHO classification (8, 9). One unstained positive-charged slide from each of 23 FFPE tissue blocks was prepared for Pan-Trk immunostaining.

### Immunohistochemistry Technique

#### IHC Protocol

4-μm FFPE tissue sections were used in the process of IHC. The IHC assay was performed using anti-Pan-Trk antibody (clone EPR#17341, rabbit monoclonal antibody, Abcam, Cat# Ab181560). The procedure was performed with the ultraView DAB detection Kit (Ventana) on a BenchMark XT automated stainer from Ventana (Tucson, AZ, United States). A protocol was established so that the entire assay procedure consisted of deparaffinization with EZ Prep at 75°^C^, heat pre-treatment in cell conditioning medium (Ag unmasking) (CC1; Ventana) for 60 min and then primary incubation for 16 min at 37°C. The antibodies were optimized using a dilution of 1:50. The slides were counterstained with hematoxylin II and bluing reagent for 16 min. After that, the slides were removed from the slide stainer and then immersed into successive alcohol buffers at different concentrations for 3 min.

#### IHC Assessment

**FIGURE 2:**
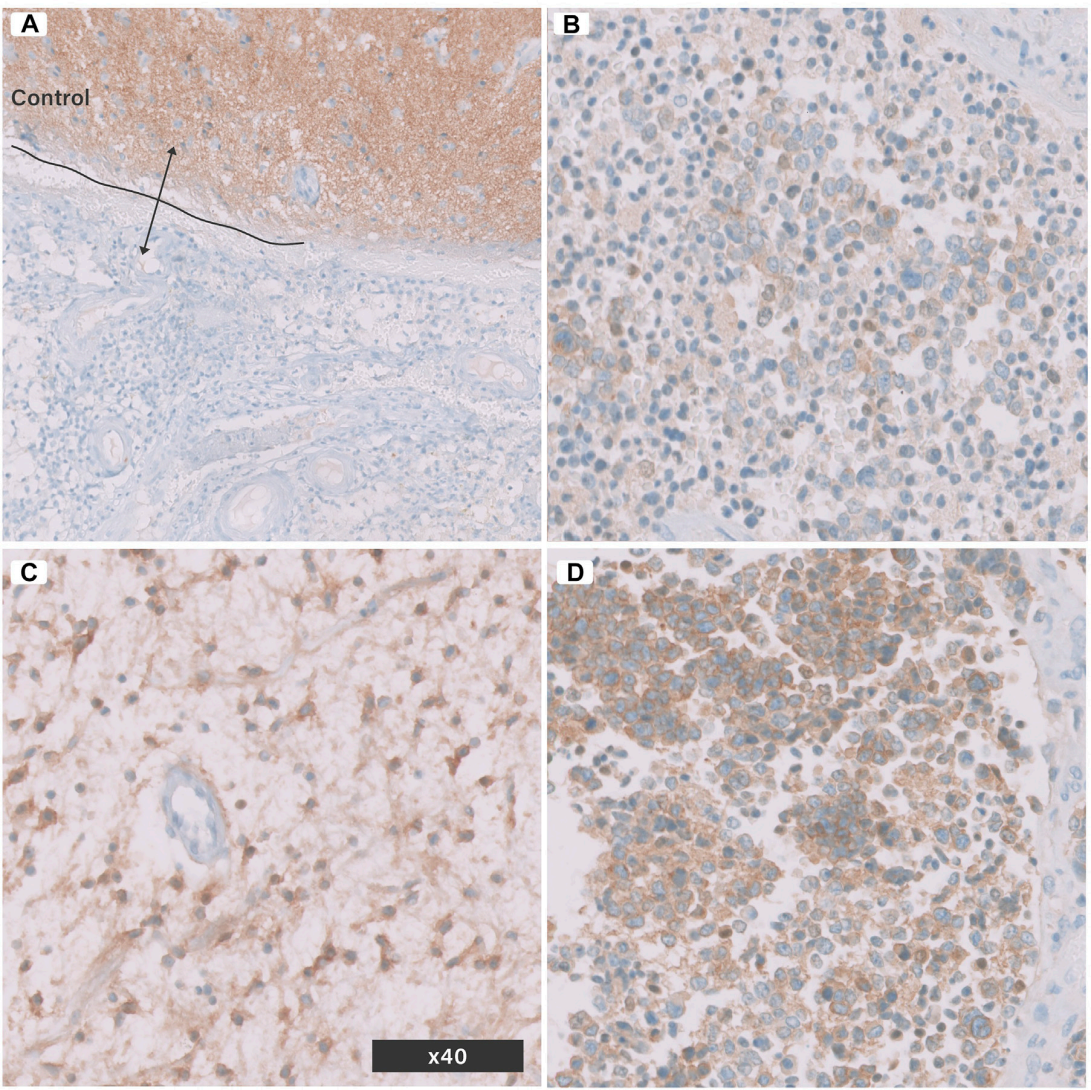
Pan-Trk Expression (nuclear or cytoplasmic) in brain tumours using IHC. **(A)** control normal brain tissue and tumour tissue negative for Pan-Trk, **(B)** focal expression, **(C)** partial expression, **(D)** diffuse expression. All images are in (×40) magnification.

Anti Pan-Trk antibody normally stains neuropil background (Figure 2A). Each tissue section was screened at a low power field (×10) using digital microscopy, and a single hot spot of a non-necrotic area was selected to count the cells manually at a high-power field (×40). Cells expressing Pan-Trk were considered positive (stained-tumour cells), while the total cells included both stained tumour cells and non-stained tumour cells. The non-stained tumour cells included lymphocytes and other types of glial cells. The labeling index was assessed using the following equation:

$$Labelling\;index\left(\%\right)=\left[\frac{\left(Pan-trk\;stained\;tumour\;cells\right)}{\left(Total\;cells\right)}\times 100\right].$$

The staining pattern was then categorized as i) diffusely expressed, ii) partially expressed, iii) focally expressed, and iv) non-expressed (Figures 2B–D; Table 2). The quantitative method used in the current study was typical of the method described by Kurdi et al (10).

**TABLE 2:** Quantitative expression of Pan-Trk in tumour cells using a digital microscope.

Expression	Labelling index (%)
No expression	0
Focal expression	>0–20
Partial expression	>20–50
Diffuse expression	>50

For statistical analysis, the scores were divided by 100.

## Next-Generation Sequencing

**FIGURE 3:**
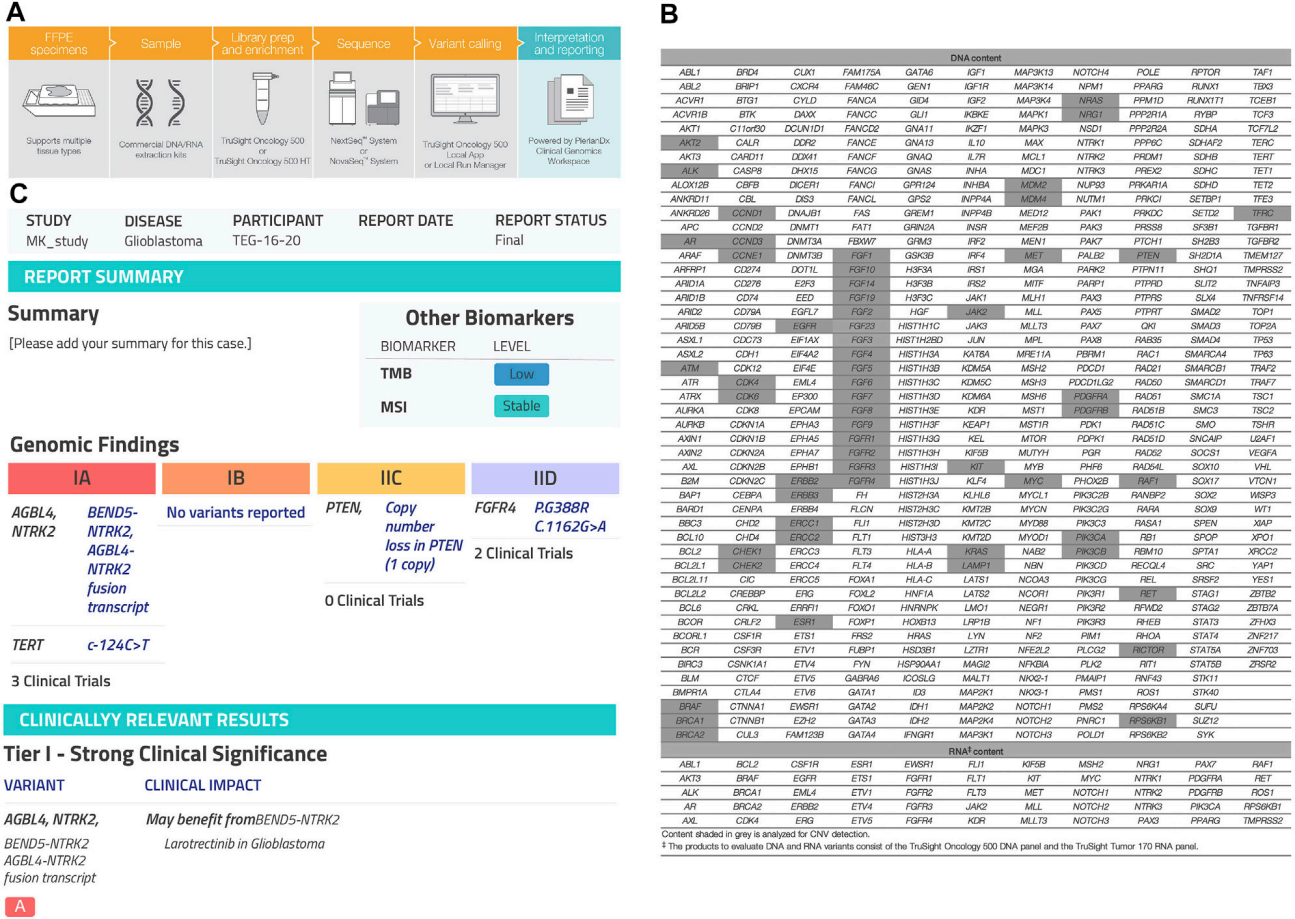
Next-generation sequencing (NGS) assay. **(A)** TruSight Oncology500 workflow from illumina integrates into lab workflows, going from nucleic acids to a variant calls in 3–4 days. (www.illumina.com/tso500), **(B)** TSO500 analyzes 500 cancer-relevant genes from both DNA and RNA in one integrated workflow. The assays assess multiple variant types (SNVs, indels, CNVs, splice variants, fusions, and emerging biomarkers that rely on analysis of multiple genomic loci, such TMB and MSI, **(C)** Clinical report generated by PierianDx after the FASTQ files and VCF being uploaded to the Clinical Genomics Workbench (PierianDx, France).

*NTRK* rearrangement was detected by DNA-based or RNA-based NGS performed at the time of research investigation. FFPE samples were collected from 23 patients diagnosed with different histopathological diagnoses. DNA and RNA were extracted using QIAMP DNA FFPE kit and RNeasy FFPE kit, respectively. DNA and RNA quality was estimated by a Nanodrop 2000 spectrophotometer (Thermo Scientific, United States), with an OD 260/280 value between 1.8 and 2.0. Qubit (Invitrogen, United States) was used to quantify nucleic acids. The minimum accepted DNA/RNA input was 50 ng. Trusight Oncology 500 (TSO500^R^) high throughoutput library preparation kit (Illumina, United States) using Nextseq 550, for screening 500 gene variants including single nucleotide variants (SNVs), fusions, splice variants, copy number variants (CNVs), microsatellite instability (MSI) and tumor mutational burden (TMB). (Figures 3A,B). An enrichment-based technology was used to perform library preparation for both DNA and RNA (www.illumina.com/tso500). Genomic DNA (gDNA) sample quality was assessed using Illumina FFPE QC. Next, gDNA was sheared to 90–250 bp. The fragmentation of gDNA was optimized using ME220 Focused-ultrasonicator (Covaris, United States). End repair and A-Tailing was performed on sheared gDNA samples. RNA sample integrity was evaluated via Agilent Technologies, 2100 Bioanalyzer, using Agilent RNA 6000 Nano kit (Agilent). RNA samples were denatured and primed to synthesize complementary DNA (cDNA). UMI1 adapters containing unique indexes were ligated to DNA fragments. Short Universal Adapters 1 (SUA1) were ligated to cDNA fragments. After that, ligated fragments were purified using sample purification beads (SPB). To allow up to eight libraries to be pooled and sequenced together, unique indexing primers were added to purified gDNA and cDNA fragments to be amplified in preparation for sample multiplexing. Following TSO 500 protocol, two hybridization steps were performed. During the first step, a pool of oligos specific to 523 genes were hybridized to DNA libraries, while a pool of oligos specific to 55 genes hybridized to RNA libraries. Then, probes hybridized to the targeted regions were captured using streptavidin magnetic beads (SMB). The second hybridization step was performed to ensure specificity of captured regions. A pool of primers was used to amplify enriched libraries. The libraries were then sequenced on the illumina Nextseq 550 platform. The run data were uploaded to the Clinical Genomics Workbench (PierianDx, France). QC analysis, mapping to hg19, variant calling, and annotation were all performed (Figure 3C). The extraction, validation, hybridization, library preparation and genomic sequencing were all performed at CAP-accredited center of excellence of genomic medicine research at King Abdulaziz University.

### Statistical Methods

The data were described as frequencies and percentages. Fisher’s exact test was used to test the significant relationship between Pan-Trk expression and *NTRK*-fusions results. *p*-value less than 0.05 was considered significant. All statistical analyses were performed using the IBM SPSS1 ver. 24 and R-Package statistical software programs (“Circlize” version is 0.4.13).

## Results

**FIGURE 4:**
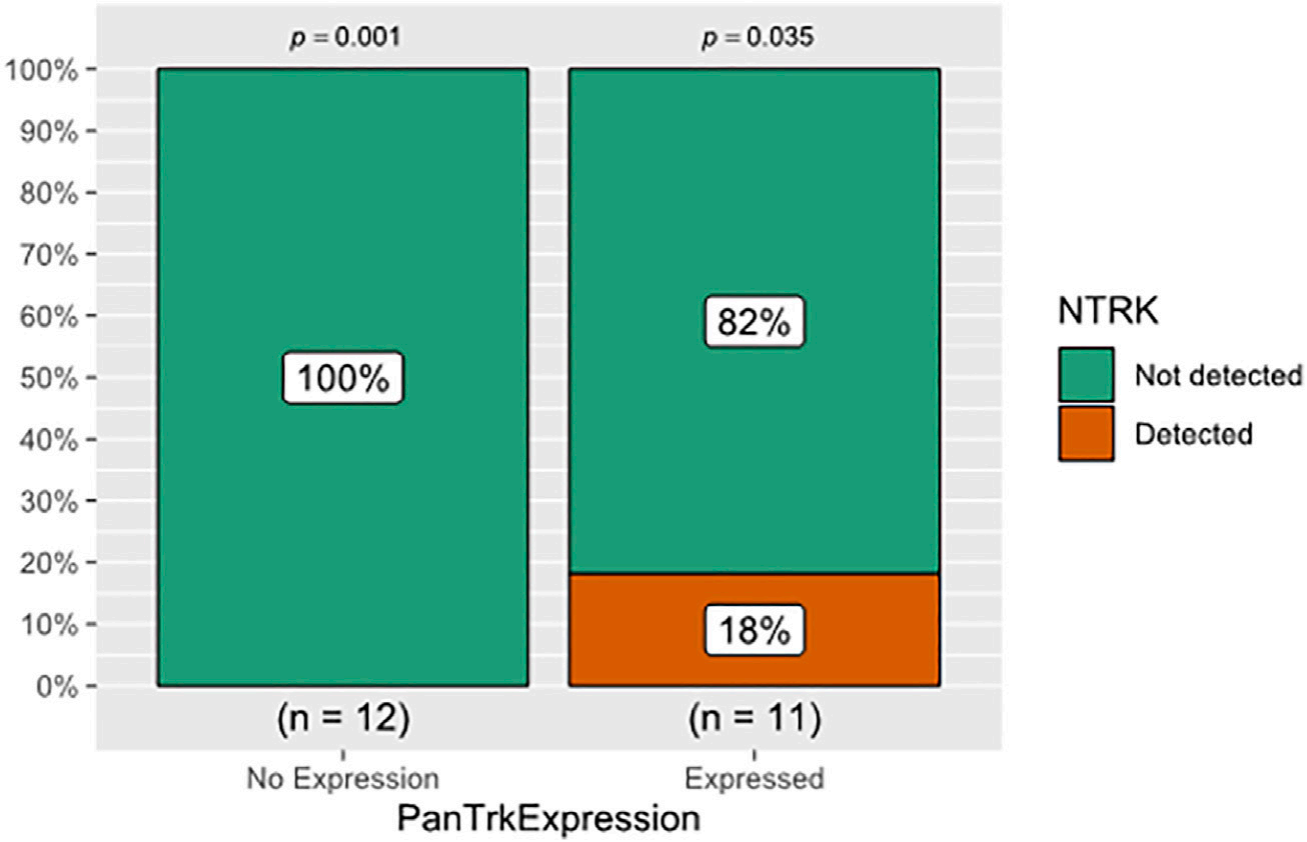
The relationship between Pan-Trk expression using IHC and NTRK-fusions detection using NGS. Pan-Trk expression was detected in 11 tumours and 12 tumours showed no Pan-Trk expression. Only two of the expressed cases (18%) were found to have *NTRK2*-fusions, and the remaining 9 Cases (82%) did not reveal any *NTRK*-fusions. The 12 cases with no Pan-Trk expression showed no *NTRK*-fusions. There was no statistically significant association between IHC and NGS in detecting *NTRK*-fusion (*p* > 0.05).

**FIGURE 5:**
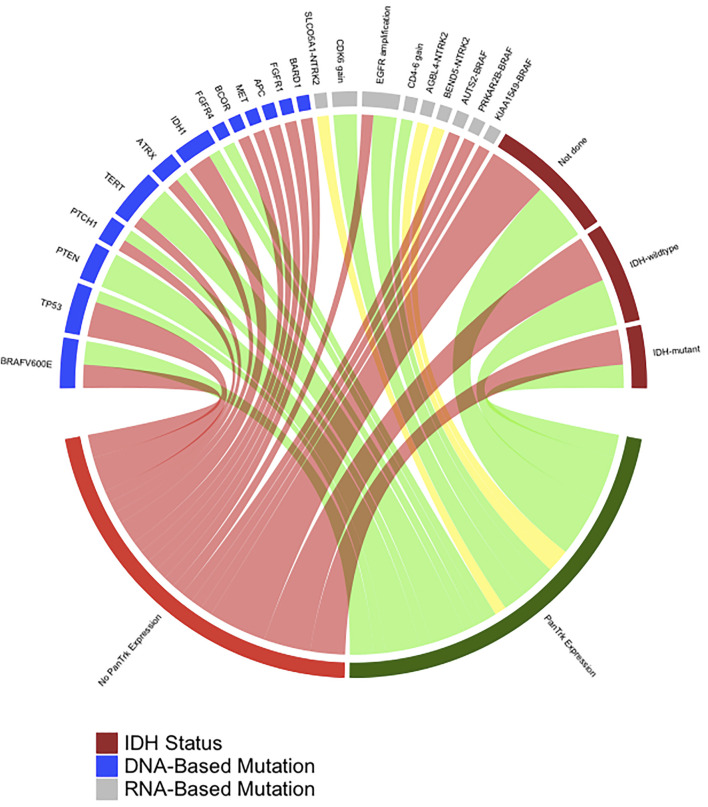
A diagram was generated by R-package, which shows the association between Pan-Trk expression with *NTRK* and non-*NTRK* fused tumours. In addition, it shows other detected DNA-based and RNA-based mutations by TruSight Onco500 through NGS.

The cohort included 23 patients diagnosed with different types of CNS tumours (pilocytic astrocytomas (n = 8), oligodendroglioma (n = 1), IDH^wildtype^ glioblastomas (n = 6), IDH^mutant^ WHO grade 4 astrocytomas (n = 4), astroblastoma (n = 1), central neurocytoma (n = 1), medulloblastoma (n = 1) and liponeurocytoma (n = 1) (Table 1). The mean age: 35.8 years (±20.7 years); seven cases were in the paediatric-age group, and 16 cases were adult; 15 males (65.2%) and eight females (34.8%). Approximately 26.1% (n = 6) of the tumours were in the frontal lobe followed by the temporal lobe (21.7%, n = 5), parietal lobe (17.4%, n = 4), posterior fossa (21.7%, n = 5), and one case for each (lateral ventricle, hypothalamic, and spinal cord) (Table 1). Pan-Trk expression was detected in 11 tumours (47.8%) and 12 tumours (52.1%) showed no Pan-Trk expression. The Pan-Trk expressed tumours (n = 11) were clustered into diffuse expression (17.4%, n = 4), partial expression (13%, n = 3) and focal expression (17.4%, n = 4). Two of these cases (18%) (liponeurocytoma, glioblastoma) with Pan-Trk expression (diffuse, partial) were found to have *NTRK2*-fusions (*SLC O 5A1-NTRK2, AGBL4-NTRK2, BEND5-NTRK2*) and these cases were adult. Additionally, one of those cases (glioblastoma) was found to have DNA-based mutations (*PTEN, TERT-promoter, and FGRF4*). On the other hand, the 9 Cases (82%) with different Pan-Trk expressions did not reveal any *NTRK*-fusions. 100% (n = 12) of the tumours with no Pan-Trk expression have shown no *NTRK*-fusions (Figure 4, 5).

There was no statistically significant association between Pan-Trk expression using IHC and *NTRK*-fusion using NGS (*p* = 0.217) (Table 3; Figure 4). This clarifies that Pan-Trk expression does not always correlate with the presence of demonstrable *NTRK*-fusions. The sensitivity of Pan-Trk IHC relative to NGS to detect *NTRK*-fusion was relatively high (100%) (two positive cases were detected by Pan-Trk IHC). However, the ability to detect negative cases by Pan-Trk IHC was observed to be 57.1%. Overall diagnostic accuracy of the PanTrk in detecting *NTRK*-fusions was 60.9% in the group with prevalence of 8.7% (Table 4).

**TABLE 3:** The relationship between Pan-Trk expression using IHC and *NTRK*-fusion detection by NGS.

Dependent: PanTrk expression		No expression	Expressed	Total	*p*-value
*NTRK-*fusion	Detected	0 (0.0)	2 (18.2)	2 (8.7)	0.217^a^
	Not detected	12 (100.0)	9 (81.8)	21 (91.3)	

a Fisher’s Exact Test.

**TABLE 4:** Sensitivity, specificity, and diagnostic accuracy of using NGS method over Pan-Trk IHC to detect *NTRK*-fusions.

NTRK-fusion	No NTRK fusion	Total
Expressed*-*PanTrk	2	11
No PanTrk expression	0	12
Total	2	23
		**Ratios**
Sensitivity		100%
Specificity		57.1%
Accuracy		60.9%
Prevalence		8.7%
Positive predictive value		18.2%
Negative predictive value		100%
Post-test disease Probability		18.02%

## Discussion

Although *NTRK*-fusions have been detected in a small number of pediatric and adult tumour types, they have also been identified in other common cancers at lower frequencies. These findings suggest that a diagnostic strategy managed by *NTRK*-fusions biological incidence may be the best effective approach to identify patients with *NTRK-*fusions. Furthermore, such *NTRK*-fusions have now been shown to be actionable genomic signatures, predicting therapeutic responses against Trk receptors, making their detection an evolving clinical priority (11).

Screening of *NTRK*-fusions is usually performed at molecular level using NGS or FISH technique (12), DNA or RNA targeted testing. However, the molecular technique is costly, time-consuming, and unavailable in most centers, and sometimes associated with sampling errors due to nucleic acid degradation. Alternatively, anti-Pan-Trk (IHC) is commonly used to examine protein expression. The clone reacts with the C-terminus of Trk-A, -B, and–C and is therefore reactive with gliomas harbouring *NTRK*-fusions. IHC is associated with limited costs and a fast turnaround time, allowing good histological correlations and protein expression validation. The major limitation is that the antibody is restricted to Trk receptors’ wildtype epitopes, thus not specific to detect *NTRK*-fusions. They do not provide additional information about the fusion partner. Instead, FISH technique was found more useful than Pan-Trk IHC as a screening tool to detect NTRK-fusion prior to RNA sequencing (12).

Pan-Trk IHC can be used as an effective screening tool for most cancers. Hechtman et al. tested 23 cases of non-CNS carcinomas with *NTRK-*fusions, in which 16 cases showed positive fusion transcript with Archer fusion and six of them were novel rearrangements (6). The 20 cases stained positively with Pan-Trk were concordant with Archer RNA, two cases with *NTRK* rearrangements showed negative in both Archer fusion and IHC. A single case of a fusion-positive colorectal carcinoma with an *ETV6-NTRK3* fusion was discordant with IHC, which showed no expression (6). This specificity and sensitivity were found low in CNS neoplasms due to the physiological expression of Pan-Trk receptors in normal CNS neuropil (Figures 2A– Control). Solomon et al. also reported an unsatisfactory specificity value of 20.8% in CNS gliomas (7). FISH showed better results than Pan-Trk in CNS tumours, particularly gliomas. RNA sequencing analyses are necessary in FISH positive cases with less than 30% positive nuclei, to avoid false positivity when scoring is close to the detection threshold (12).

Our results showed that Pan-Trk expression was detected in 11 tumours (47.8%) and 12 tumours (52.1%) showed no Pan-Trk expression. Out of the 11 cases, nine cases (82%) did not reveal any *NTRK*-rearrangement, while two cases were found to have *NTRK2*-fusions (*SLC O 5A1-NTRK2, AGBL4-NTRK2, BEND5-NTRK2*) (Figures 4, 5). The rest of the 12 cases with no Pan-Trk expression showed no *NTRK*-fusion. Our results also showed that NGS is the best molecular method to detect *NTRK*-fusions with 100% specificity compared to Pan-Trk IHC, which showed low specificity (Table 4). This is likely related to the normal physiological expression of Trk protein receptors in normal brain tissue, which may falsely predict *NTRK*-fusions. Moreover, using TruSightOnco500 platform replaced IHC and other molecular methods to detect a wide range of DNA-based and RNA-based mutations.

Finally, one limitation must be acknowledged in our study is, that the total number of cases analyzed for *NTRK-*fusions and Pan-Trk IHC is relatively low. Despite this limitation and to our best knowledge, this is the first study, globally and particularly in Saudi Arabia, that investigate the tyrosine kinases biomarkers in different CNS tumours, reflecting the accuracy of diagnostic technique on patient management.

## Conclusion

Pan-Trk IHC is not a suitable tissue-efficient biomarker to screen for *NTRK*-fusions in CNS tumours. Its usage should be with extreme caution, and its confirmation by other techniques is warranted. RNA-based NGS sequencing should be used as an alternative method to detect *NTRK*-fusions. TruSightOnco500 is a wide-genomic platform that can replace IHC and other molecular techniques to screen for DNA and RNA-based mutation using FFPE tissue.

## Data Availability

The original contributions and data presented in the study are available upon request from the corresponding author.
